# Perfluorooctanoic acid, perfluorobutanoic acid, and undecafluoro-2-methyl-3-oxahexanoic acid disrupt neurotransmitter release and cholinesterase activity

**DOI:** 10.1016/j.chemosphere.2025.144678

**Published:** 2025-09-11

**Authors:** Precious C. Obiako, Bryan A. Taylor, Jonathan A. Clinger, Christie M. Sayes

**Affiliations:** a Department of Environmental Science, Baylor University, Waco, TX, 76798, USA; b Department of Chemistry and Biochemistry, Baylor University, Waco, TX, 76798, USA

**Keywords:** Binding sites, Cholinesterase, Toxicity, Environmental, Molecular docking

## Abstract

Perfluoroalkyl substances (PFAS) induced neurotoxicity is an emerging concern, with evidence increasingly linking it to the dysregulation of neurotransmission pathways. This study investigates the effects of three different PFAS, namely perfluorooctanoic acid (PFOA), perfluorobutanoic acids (PFBA), and undecafluoro-2-methyl-3-oxahexanoic acid (GenX), on catecholamine neurotransmitter release (dopamine, noradrenaline, and adrenaline) and neurotransmission-regulating enzymes (acetylcholinesterase (AChE) and butyrylcholinesterase (BChE)). Neurotransmitter release assays revealed that PFAS exposure in neuronal cells increased dopamine and noradrenaline levels but reduced adrenaline levels. Cholinesterase activity was assessed in cell-free conditions and human neuronal cells (SH-SY5Y), revealing PFAS-induced enzymatic perturbations characterized by decreased AChE activity and increased BChE activity. Fluorescence and synchronous fluorescence spectroscopy highlighted differential enzyme-PFAS interactions, which were corroborated by molecular docking results and revealed compound-specific interactions with amino acid residues. Of the substances tested, PFOA exhibited the strongest binding interaction. ^19^F nuclear magnetic resonance (NMR) spectroscopy also demonstrated differential binding interactions among the PFAS and AChE or BChE. This study provides mechanistic insights into PFAS-induced neurotoxicity, highlighting their potential to disrupt neurotransmitter and enzymatic dynamics crucial for neurological health.

## Graphical Abstract

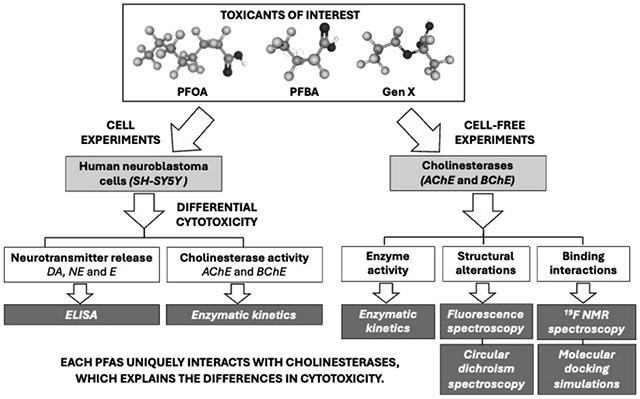

## 1. Introduction

Per- and polyfluoroalkyl substances (PFAS) are synthetic organic chemicals defined by their strong carbon-fluorine bonds, unique physicochemical properties, and remarkable stability (Solan et al., 2022; Starnes et al., 2022). These attributes have driven their widespread use across diverse industrial processes and consumer products, resulting in their pervasive presence (Singh et al., 2023; Dirani et al., 2024). The persistence of PFAS and their resistance to degradation promote bioaccumulation in various environmental matrices and biological systems. This has raised substantial scientific and regulatory concerns regarding their potential human and ecological health impacts (Obiako et al., 2024).

The development of perfluorinated alkyl substances began with long-chain compounds, with perfluorooctanoic acid (PFOA) and perfluorooctanesulfonic acid (PFOS) being prominent examples (Chambers et al., 2021). The industry initiated a strategic shift toward shorter-chain alternatives as evidence accumulated regarding the environmental persistence and toxicological properties of the long-chain compounds. This transition introduced compounds such as perfluorobutanoic acid (PFBA) and undecafluoro-2-methyl-3-oxahexanoic acid (HFPO-DA, marketed under the trade name GenX), which were developed to mitigate the environmental and health concerns associated with long-chain PFAS (Calafat et al., 2019; Kwiatkowski et al., 2020). Nevertheless, recent scientific investigations suggest these short-chain alternatives may exhibit toxicological impacts comparable to, or in some cases exceed, those observed with their long-chain predecessors (Obiako et al., 2024; Chambers et al., 2021; Zheng et al., 2023).

Several studies have demonstrated that certain PFAS can cross the blood-brain barrier and accumulate in the brain tissues of humans and animals following environmental or experimental exposure (Starnes et al., 2022; Xie et al., 2022; Di Nisio et al., 2022; Kawabata et al., 2017). It has been suggested that the unique surfactant properties of these chemicals may enhance their ability to disrupt cell membranes, facilitating their entry into the brain (Brown-Leung et al., 2022). Notably, some PFAS have been shown to compromise the neural protective features of astrocytes and endothelial cells within the blood-brain barrier, thereby increasing permeability and susceptibility to xenobiotic infiltration (Yu et al., 2020; Wang et al., 2018).

Although extensive research has characterized various toxicological effects of PFAS exposure, a notable gap remains in understanding their neurotoxic potential. Limited studies have investigated the effects of PFOA and PFOS on the nervous systems of laboratory animals. Specifically, some rodent studies have highlighted cognitive function deficiencies following PFOA and PFOS exposure (Starnes et al., 2022; Shi et al., 2022). Epidemiological research has linked PFAS exposure to adverse neurological health outcomes, including attention-deficit hyperactivity disorder (ADHD) and autism spectrum disorder (ASD) (Currie et al., 2024; Kim et al., 2023). Additionally, exposure to these persistent chemicals has been associated with reduced cognitive function, evidenced by lower intelligence quotient (IQ) scores and an increased risk of neurodegenerative conditions such as Parkinson’s and Alzheimer’s disease (Wang et al., 2015; Delcourt et al., 2024).

PFAS-induced neurotoxicity is increasingly associated with perturbations in neurotransmission pathways, including alterations in neurotransmitter levels and neurological enzyme function (Brown-Leung et al., 2022; Mulkiewicz et al., 2007; Foguth et al., 2020a, 2020b; Eggers Pedersen et al., 2015). Neurotransmitters and neurological enzymes are pivotal in transmitting signals and regulating behavior, cognition, and overall neurological health (Teleanu et al., 2022; Singh et al., 2024; Gajendra et al., 2024). Mounting evidence suggests that exposure to individual PFAS, such as PFOA, PFOS, PFBS, and PFAS mixtures, can disrupt neurotransmitter homeostasis and associated metabolic processes. PFAS exposure has also been shown to modulate the function and expression of neurotransmitter receptors and transporters, leading to impaired neural communication (Brown-Leung et al., 2022; Foguth et al., 2020a, 2020b). For instance, Nanyang et al. observed elevated dopamine and serotonin concentrations, accompanied by decreased levels of norepinephrine and glutamate, in the brains of PFOA-exposed mice (Yu et al., 2016). In contrast, a study conducted using an amphibian model (Northern leopard frogs) demonstrated significant dopaminergic depletion following exposure to PFOA and PFOS (Foguth et al., 2019), suggesting species-specific variability in the neurotoxic effects of PFAS.

In addition to their effects on neurotransmitters, PFAS compounds have been implicated in disrupting cholinesterase activity, a key enzymatic pathway critical for maintaining neural homeostasis. Eggers Pedersen et al. reported decreased acetylcholinesterase (AChE) activity with increasing PFAS concentrations in specific polar bears’ brain regions (Eggers Pedersen et al., 2015). Similarly, Mulkiewicz et al. observed reduced AChE activity in leukemia (IPC-81) and glioma (C6) rat cell lines, as well as in bacterial culture, following individual exposure to five long-chain PFAS compounds (Mulkiewicz et al., 2007).

Our previous studies have shown that PFOA and PFBA significantly perturb the function of enzymes essential for antioxidant defense and metabolic processes in a cell-free model and human neuronal cells (SH-SY5Y), resulting in oxidative stress and metabolic dysregulation (Obiako et al., 2023, 2024).

This study aimed to investigate the effect of PFAS exposure on neurotransmission pathways. Utilizing the human neuroblastoma SH-SY5Y cell line as an experimental model, this study examines the neurotoxicological effects of three PFAS compounds: perfluorooctanoic acid (PFOA), perfluorobutanoic acid (PFBA), and undecafluoro-2-methyl-3-oxahexanoic acid (GenX) on catecholamine neurotransmitter release and cholinesterase function, specifically acetylcholinesterase (AChE) and butyrylcholinesterase (BChE). To further elucidate the mechanistic underpinnings of PFAS-induced perturbations of cholinesterase activity, biochemical analyses were performed under cell-free conditions to assess enzyme kinetics, binding interactions, and structural alterations. This approach provides molecular-level insights for understanding PFAS-mediated disruption of neural signaling pathways.

## 2. Materials and methods

### Chemicals and reagents.

Perfluorooctanoic acid (PFOA, 95 %), heptafluorobutyric acid (HFBA, 98 %), undecafluoro-2-methyl-3-oxahexanoic acid (GenX, 97 %), acetylcholinesterase from *Electrophorus electricus* (electric eel) with molecular weight of 280 kDa and butyrylcholinesterase from equine serum with molecular weight of 440 kDa were purchased from Sigma-Aldrich (St. Louis, MO, USA). Acetylthiocholine Iodide, Butyrylthiocholine iodide, 5,5′-dithiobis(2-nitrobenzoic acid), Dulbecco’s Modified Eagle Medium (DMEM), fetal bovine serum (FBS), penicillin-streptomycin solution, and phosphate-buffered saline (PBS) were all acquired from Thermo Fisher Scientific (Waltham, MA, USA). The physicochemical characteristics of the PFAS used in this study are shown in Table S1.

### Cell culture.

SH-SY5Y neuroblastoma cells (CRL-2266) were sourced from the American Type Culture Collection (ATCC, Manassas, VA, USA). The cells were grown as a monolayer in DMEM supplemented with 10 % fetal bovine serum (FBS) and a 1 % penicillin-streptomycin antibiotic solution, following the ATCC guidelines (Obiako et al., 2024). Cultures were maintained at 37 °C with 5 % CO_2_ in a humidified, air-jacketed incubator.

### Neurotransmitter release assay.

SH-SY5Y cells were seeded at a density of 2.0 × 10^6^ cells/well in 6-well plates and incubated overnight to ensure optimal adherence. Following adherence, the cells were treated with 1 μg/mL of PFOA, PFBA, or GenX for 24 h post-exposure; the cells were stimulated with a 50 mM potassium chloride (KCl) solution to induce depolarization (Kost et al., 2011). Supernatants were collected, and the levels of dopamine, noradrenaline, and adrenaline were quantified using a 3-CAT high-sensitivity ELISA kit (Rocky Mountain Diagnostics, Colorado Springs, CO, USA) according to the manufacturer’s protocol.

### Cholinesterase activity.

Acetylcholinesterase (AChE) and butyrylcholinesterase (BChE) catalyze the hydrolysis of acetylcholine and butyrylcholine, respectively, into their corresponding acids (acetic acid or butyric acid) and choline, critical processes in neurotransmitter regulation (Colovic et al., 2013). These enzymatic reactions were monitored using Ellman’s method, where an alternative substrate (acetylthiocholine for AChE or butyrylthiocholine for BChE) was used. In the modified method, the hydrolysis product, choline, reacts with 5, 5′-dithiobis(2-nitrobenzoic acid) (DTNB) to form 5-thio-2-nitrobenzoate (TNB), a yellow-colored compound that is measurable at 412 nm (Pohanka et al., 2011; Ellman et al., 1961).

### Mammalian cell-based assay.

AChE and BChE activities in live SH-SY5Y cells were assessed using an established protocol (Lee et al., 2022; Onder et al., 2022; Ehrich et al., 1997). SH-SY5Y cells were seeded at a density of 5.0 × 10^4^ cells/well in 96-well plates and incubated overnight to ensure optimal adherence. PFAS solutions were prepared by dissolving directly in culture medium. Untreated control cells were maintained in the same culture medium without PFAS and incubated under identical conditions. Subsequently, the cells were exposed to different concentrations of PFOA, PFBA, or GenX (0.01, 0.1, and 1 μg/mL) and incubated for 24 h. The 24-h exposure time point was chosen as it is commonly used for acute toxicity screening in toxicology, allowing sufficient time for cellular uptake and responses to chemical exposure. Following the exposure period, the assay medium was removed, and the cells were incubated with a reaction mixture consisting of either acetylthiocholine (1 mM; for AChE activity) or butyrylthiocholine (1 mM; for BChE activity) and 5,5′-dithiobis(2-nitrobenzoic acid) (0.5 mM; DTNB) in potassium phosphate buffer (0.1 M; pH 8.0) at 25°C, in a total volume of 200 μL per well. AChE and BChE activity were monitored by measuring the absorbance at 412 nm at 5-min intervals for 60 min using a Biotek Synergy H1 spectrometer (Agilent Technologies, Santa Clara, CA, USA).

### Cell-free assay.

In this context, "cell-free" refers to assays performed without the use of mammalian cells (or any other type of cell). Instead, commercially sourced enzymes were employed to investigate specific biochemical endpoints under controlled experimental conditions. PFAS solutions were prepared in phosphate buffer. Controls were run in parallel without the addition of PFAS under identical conditions. Briefly, commercially available AChE and BChE (0.1 unit/mL) were preincubated with PFOA, PFBA, or GenX at pH 8.0 and 25 °C for 15 min. A 15-min pre-incubation period was selected based on our preliminary experiments and literature reports that utilize 10–15 min and demonstrate maximal inhibition of several cholinesterase inhibitors within that timeframe (Loewenthal et al., 2022). The units are based on the enzyme activity level provided by the manufacturer. PFAS concentrations are described in Table 1.

The enzymatic reaction was initiated by adding DTNB and the corresponding substrate (acetylthiocholine for AChE or butyrylthiocholine for BChE). Absorbance at 412 nm was recorded at 10-s intervals over 5 min. The slope of the absorbance versus time, indicative of the reaction rate or the rate of TNB formation, was used to quantify AChE and BChE activity in both cell-based and cell-free assays (Figures S1-S4). Relative enzyme activity was determined by comparing the reaction rates of treated samples to those of untreated controls.

### Spectroscopic analysis of cholinesterase-PFAS interactions.

AChE and BChE were individually incubated with 0.01–10 μg/mL of PFOA, PFBA, and GenX as separate preparations in potassium phosphate buffer (0.1 M, pH 8.0) at 25 °C. Following incubation, fluorescence, synchronous fluorescence, and circular dichroism spectroscopy were employed to evaluate alterations in the spectroscopic properties of the cholinesterases. The absorbance measurements, as shown in Fig. S6 and S7, confirm that the enzyme concentrations remained consistent during the experiments.

### Fluorine-19 nuclear magnetic resonance spectroscopy.

^19^F NMR analysis was performed using a Bruker 600 MHz NMR spectrophotometer (Bruker BioSpin, Rheinstetten, Germany). Spectral acquisition was performed with 200 scans and a relaxation delay of 15 μs. For the analysis, PFAS concentrations were held constant at 1 mg/mL by weight. AChE and BChE concentrations varied at 0.5 mg/mL and 0.2 mg/mL, corresponding to PFAS:enzyme concentration ratios of 2:1 and 5:1, respectively. These ratios were selected based on preliminary findings and prior studies indicating that PFAS must be in excess relative to protein to observe significant chemical shift perturbations (CSPs) (Fedorenko et al., 2021; D’Eon et al., 2010). The samples were prepared in PBS and 10 % D_2_O (essential for NMR signal locking and calibration) and left to incubate for 30 min to facilitate potential binding or interactions between PFAS:AChE and PFAS:BChE, before being transferred to 5 mm NMR tubes for spectral acquisition.

### Fluorescence and synchronous fluorescence spectroscopy.

Fluorescence measurements were conducted using a Cary Eclipse fluorescence spectrometer (Agilent Technologies, Santa Clara, CA, USA). All experiments were performed in a 1.0 cm path-length quartz cuvette. Both excitation and emission slit widths were set to 5.0 nm. The scan speed was maintained at 1200 nm/min, and the photomultiplier tube (PMT) voltage was set to 600 V. The excitation wavelength was fixed at 280 nm, and emission was recorded over 290–450 nm.

To obtain the changes around individual tyrosine (Tyr) and tryptophan (Trp) residues in the enzymes, the synchronous fluorescence spectra were recorded from 250 to 350 nm at fixed 15 nm intervals and 200–350 nm at fixed 60 nm intervals between the excitation and emission wavelength both with a slit width of 2.5 nm, respectively (Qin et al., 2011; Magdy et al., 2022; Fan et al., 2024).

### Circular dichroism spectroscopy.

Circular dichroism (CD) spectra were acquired utilizing the Jasco Circular Dichroism Spectropolarimeter (Easton, Maryland, USA). Spectra were collected across the 190–260 nm wavelength range using a 10 mm quartz cuvette and a scan speed of 100 nm/min under a nitrogen atmosphere.

### Molecular docking.

Crystal structures of human acetylcholinesterase (4EY7) and human butyrylcholinesterase (4TPK) were obtained from the Protein Data Bank (PDB) for docking. Each structure was prepared for docking using Autodock Tools (ADT) (Morris et al., 2009). Crystallographic waters and heteroatoms incompatible with Vina were removed. Polar hydrogens and Kollman charges were added, and the charge deficit was spread over all atoms. Atoms were assigned as AD4 type, and the edited structure was saved as a PDBQT file. Ligand sdf files were obtained from PubChem, and Avogadro was used to optimize ligand geometry and add hydrogens. Sdf files were converted to pdbqt files using the mk_prepare_ligand script, part of the ADT suite. Grid boxes of 50 Å in each dimension with a spacing of 0.5 Å were manually positioned using ADT to cover one monomer of the protein of interest, serving as the search space for the docking. All docking was performed using AutoDock Vina with the Vina force field (Eberhardt et al., 2021; Trott et al., 2010). All final structures were rendered using PyMOL (Schrodinger, 2010).

### Statistical Analysis.

All experiments were conducted in triplicate (technical replicates) and repeated in three independent experiments (biological replicates). Statistical analyses were performed using GraphPad Prism (Version 10.4.0). The normality of data distributions was assessed using the Shapiro–Wilk test. For the neurotransmitter release assays, Welch’s *t*-test was used to compare PFAS-treated groups to untreated controls. For enzyme activity assays, analysis of covariance (ANCOVA) was employed to compare the reaction rates (slope of absorbance vs. time) between treated and control samples. Differences were considered statistically significant at p < 0.05.

## 3. Results

### Neurotransmitter release assay.

**Fig. 1.**
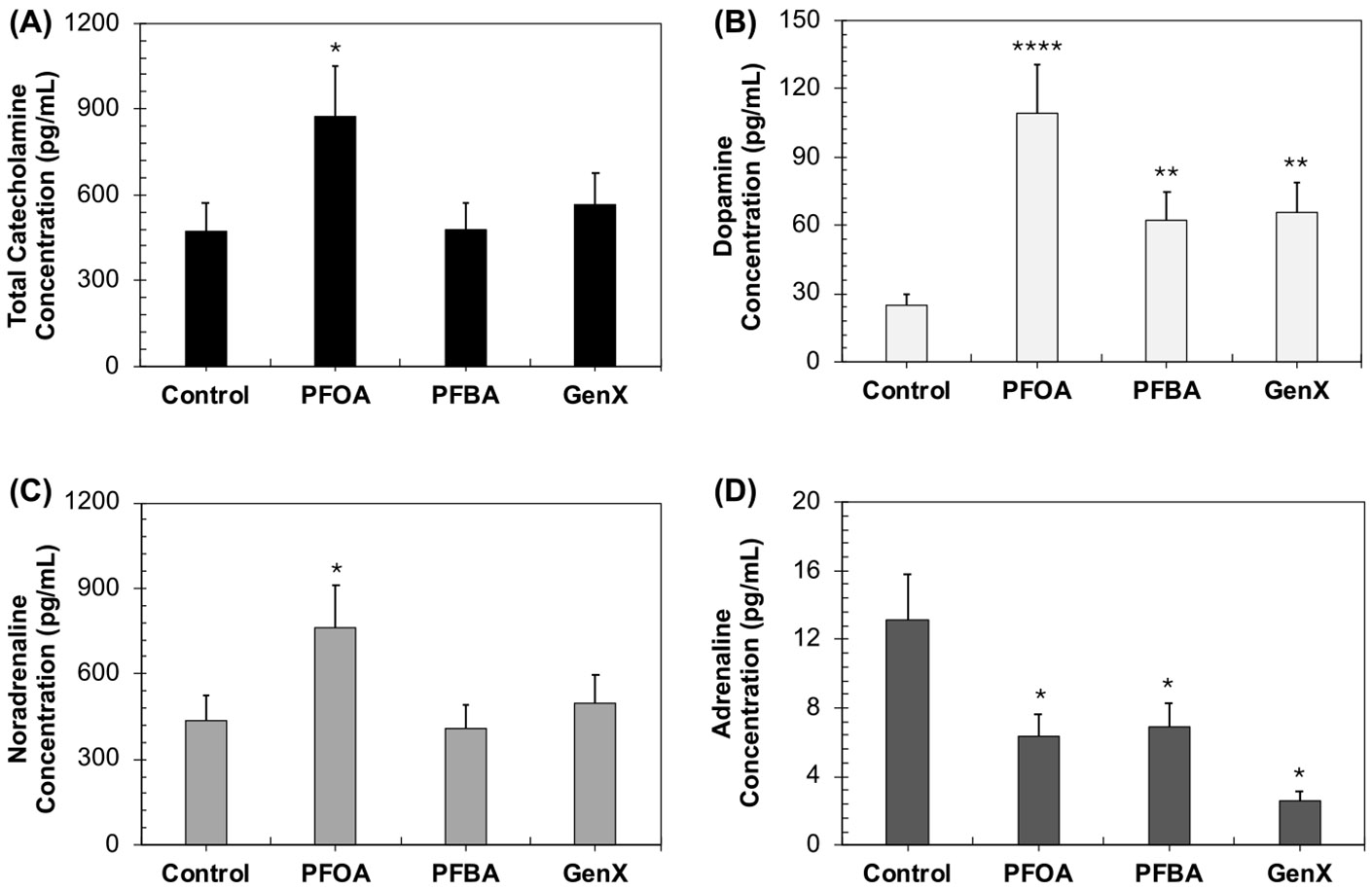
Catecholamine release from SH-SY5Y cells after 24-h exposure to 1 μg/mL PFOA, PFBA, and GenX. (A) (A) Total catecholamines that include dopamine, noradrenaline, and adrenaline; (B) dopamine; (C) noradrenaline; and (D) adrenaline levels were quantified using ELISA after depolarization of SH-SY5Y cells with 50 mM KCl, showing differential modulation by PFAS exposure. Asterisks indicate statistical significance relative to untreated controls: *P < 0.05, **P < 0.01, ****P < 0.0001.

The effects of 24-h exposure to 1 μg/mL of PFOA, PFBA, and GenX on neurotransmitter release from SH-SY5Y cells are shown in Fig. 1. Analysis of total catecholamine levels revealed a statistically significant increase only in PFOA-treated cells. In contrast, exposure to PFBA and GenX did not significantly alter catecholamine release compared to the control group (Fig. 1A). Examining individual catecholamines, PFOA induced the most pronounced increase in dopamine release; however, PFBA and GenX also caused statistically significant increases in dopamine release compared to controls (Fig. 1B). A consistent pattern was observed with noradrenaline, where PFOA exposure led to a significant increase in noradrenaline release, while PFBA and GenX had no significant effects (Fig. 1C). Adrenaline release demonstrated a contrasting trend. Exposure to PFOA, PFBA, and GenX resulted in a statistically significant decrease in adrenaline levels released from SH-SY5Y cells, with GenX causing the most pronounced reduction among the three compounds (Fig. 1D).

### Cholinesterase activity in SH-SY5Y cells.

**Fig. 2.**
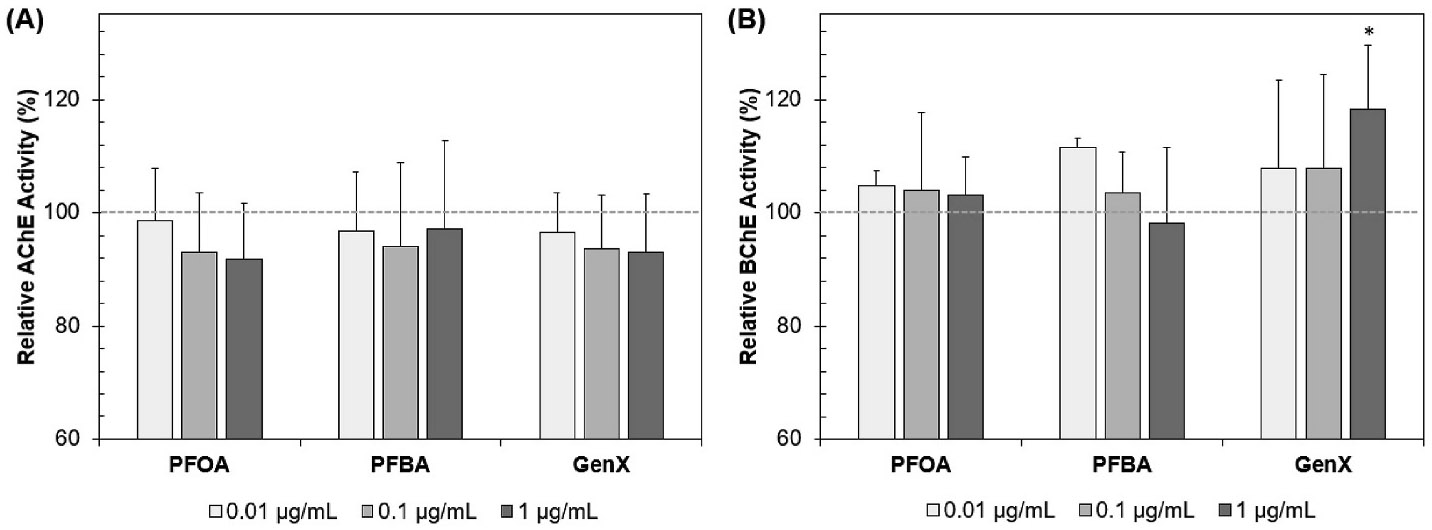
Enzyme activity of cholinesterases following 24-h exposure of SH-SY5Y cells to PFOA, PFBA, and GenX. The horizontal dotted line represents the enzyme activity of untreated cells (i.e., the control group). (A) Acetylcholinesterase (AChE) activity and (B) butyrylcholinesterase (BChE) activity were quantified and normalized to the activity of untreated control samples. Asterisks indicate statistical significance relative to untreated controls: *P < 0.05.

The activity of acetylcholinesterase (AChE) and butyrylcholinesterase (BChE) in SH-SY5Y cells after 24 h of exposure to varying concentrations of PFOA, PFBA, and GenX is shown in Fig. 2. A dose-dependent decrease in AChE activity was observed for PFOA and GenX treatments. In contrast, PFBA exhibited a reduction in AChE activity that did not follow a clear dose-dependent pattern. (Fig. 2A). However, this reduction was not statistically significant, indicating that PFAS exposure at the tested concentrations may not strongly impair AChE activity in SH-SY5Y cells under these conditions.

In contrast, BChE activity exhibited a different trend (Fig. 2B). While the overall pattern suggested an increase in enzyme activity in cells treated with PFAS, these changes were not statistically significant except in the case of cells exposed to 1 μg/mL GenX, which showed a statistically significant increase in BChE activity. This suggests a potential differential sensitivity of BChE to GenX compared to the other PFAS compounds tested.

### Cholinesterase activity in cell-free conditions.

**Fig. 3.**
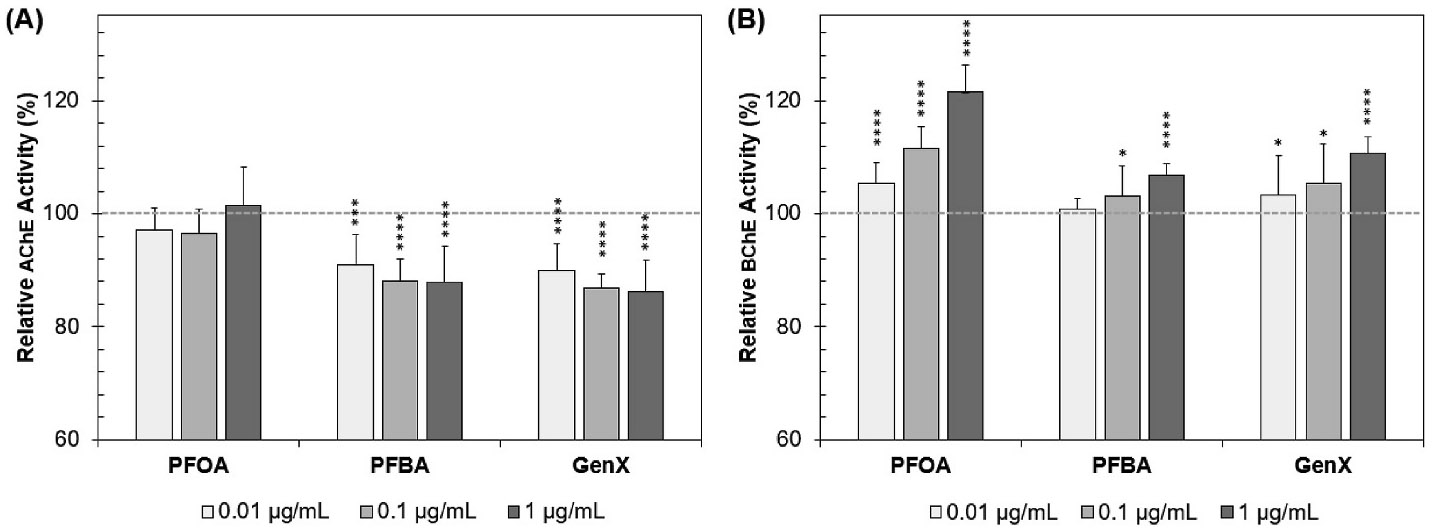
Cholinesterase activity in cell-free conditions following PFOA, PFBA, and GenX treatment. The horizontal dotted line represents the enzyme activity of untreated samples (i.e., the control group). (A) Acetylcholinesterase (AChE) and (B) butyrylcholinesterase (BChE) activities were measured and normalized to untreated controls. Asterisks indicate statistical significance compared to controls: *P < 0.05, ***P < 0.001, ****P < 0.0001.

The effects of PFOA, PFBA, and GenX on AChE and BChE activities were evaluated in cell-free enzymatic assays (Fig. 3). AChE activity remained unchanged relative to the untreated enzyme samples across all tested concentrations of PFOA. In contrast, PFBA and GenX treatments resulted in a marked and statistically significant reduction in AChE activity. At the highest tested concentration (1 μg/mL), PFBA decreased AChE activity to approximately 88 %, while GenX reduced activity to about 86 % (Fig. 3A).

For BChE activity, exposure to all three PFAS compounds (PFOA, PFBA, and GenX) significantly increased enzyme activity relative to the untreated enzyme samples. Among the treatments, PFOA exhibited the most pronounced effect, with BChE activity increasing up to 121 % at the highest tested concentration in an apparent dose-dependent manner. PFBA and GenX also enhanced BChE activity, with maximum increases of approximately 106 % and 110 %, respectively (Fig. 3B).

### ^19^F NMR spectra.

**Fig. 4.**
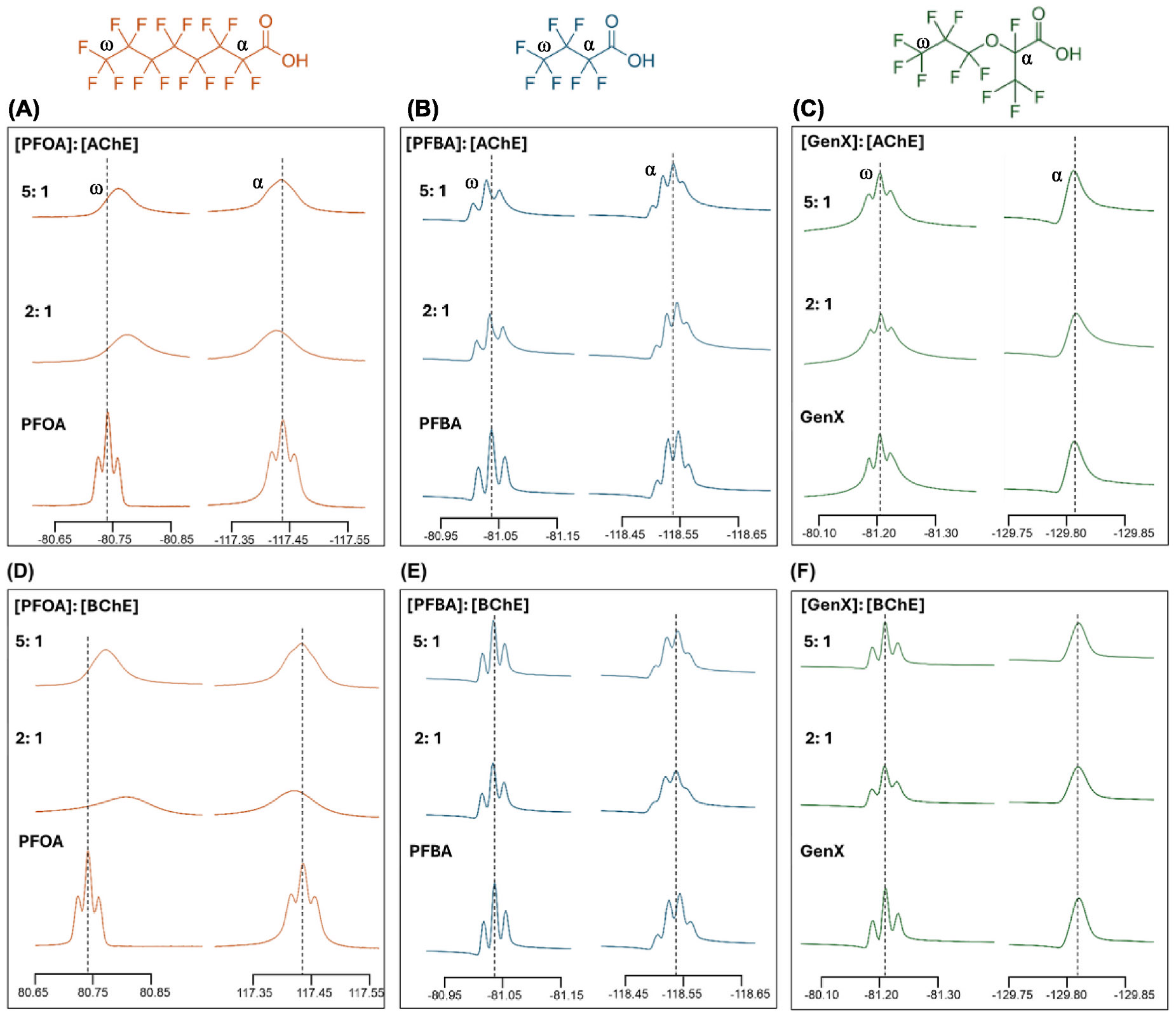
Selected fluorine nuclear magnetic resonance (^19^F NMR) peaks highlighting the characteristic R–CF_3_ (tail) and R–CF_2_–R (head, for PFOA and PFBA) or R-CF-R (head, for GenX) regions of perfluorinated compounds. (A–C) Spectra of PFOA, PFBA, and GenX in the absence and presence of acetylcholinesterase (AChE). (D–F) Spectra of PFOA, PFBA, and GenX in the absence and presence of butyrylcholinesterase (BChE). Experiments were conducted using 1 mg/mL perfluorinated compounds in 10 % D_2_O and PBS at pH 7.4. Alterations in peak positions, intensities, and broadening suggest potential binding interactions and changes in the local chemical environment caused by the interaction between the perfluorinated compounds and the cholinesterase enzymes.

The ^19^F NMR spectra of PFOA, PFBA, and GenX were consistent with those published in the scientific literature (Fig. S5). Significant changes in ^19^F chemical shifts, peak broadening, and reductions in peak intensity were observed for both the α and ω groups of PFOA upon treatment with AChE and BChE, suggesting the formation of a complex between PFAS and the enzymes (Fig. 4). This effect is more noticeable in the 2:1 PFAS:enzyme concentration ratio. There were minimal or no changes in the PFBA and GenX spectral peaks under similar treatments with AChE and BChE.

### Fluorescence and Synchronous Fluorescence Spectroscopic Analysis.

**Fig. 5.**
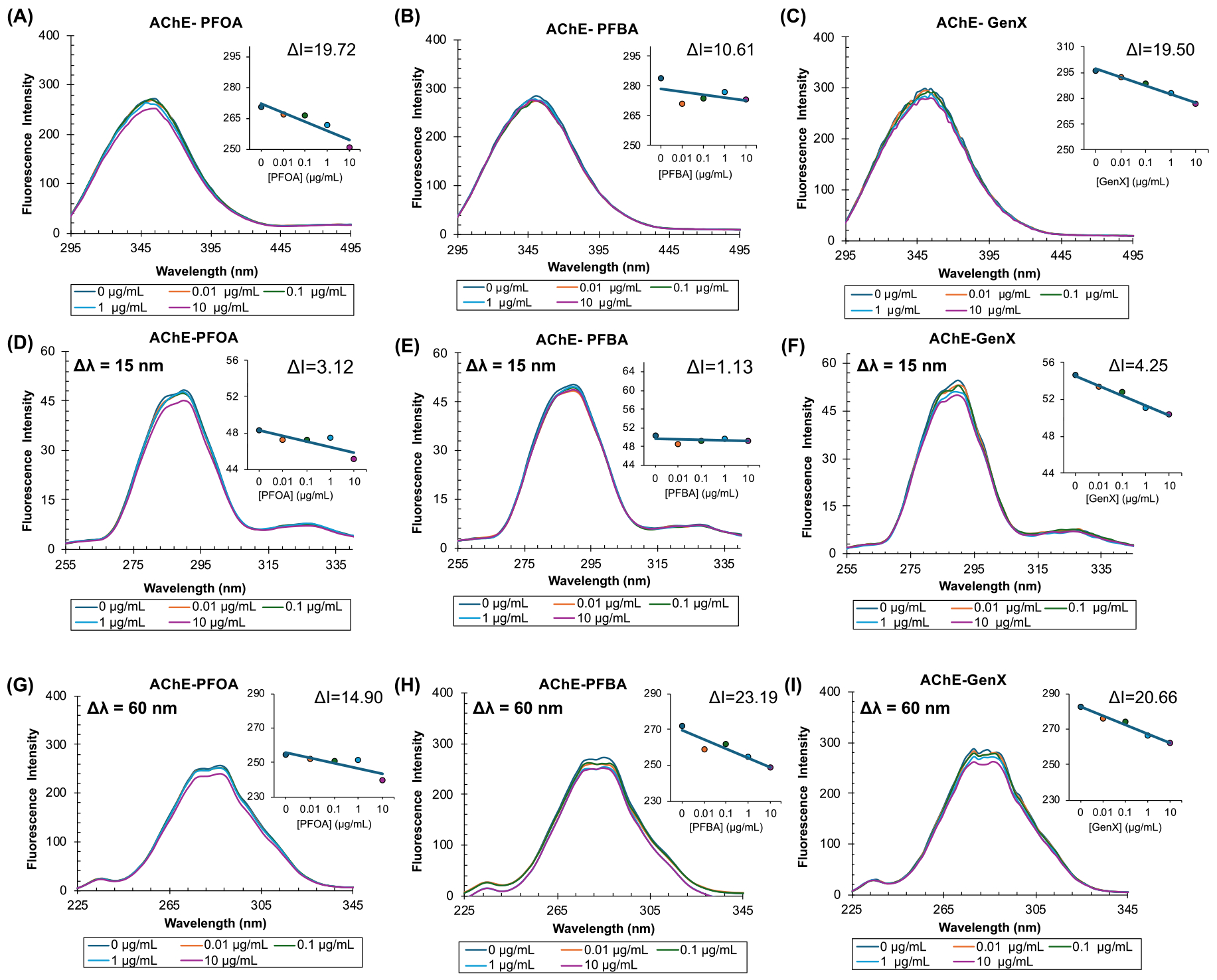
Fluorescence and synchronous fluorescence spectra of acetylcholinesterase (AChE) in the absence and presence of PFAS. (A–C) Fluorescence emission spectra (*λ*_ex_ = 280 nm; *λ*_em_ = 356 nm) of AChE treated with (A) PFOA, (B) PFBA, and (C) GenX. (D–F) Synchronous fluorescence spectra (*λ*_ex_ = 250–350 nm (Δλ = 15 nm); *λ*_em_ = 290 nm) of AChE in the presence of (D) PFOA, (E) PFBA, and (F) GenX. (G–I) Synchronous fluorescence spectra (*λ*_ex_ = 200–350 nm (Δλ = 60 nm); *λ*_em_ = 287 nm) of AChE in the presence of (G) PFOA, (H) PFBA, and (I) GenX. Insets display scatter plots of fluorescence intensity as a function of PFAS concentration. Experiments were conducted at 25 °C and pH 8.0, with an AChE concentration of 0.2 mg/mL.

**Fig. 6.**
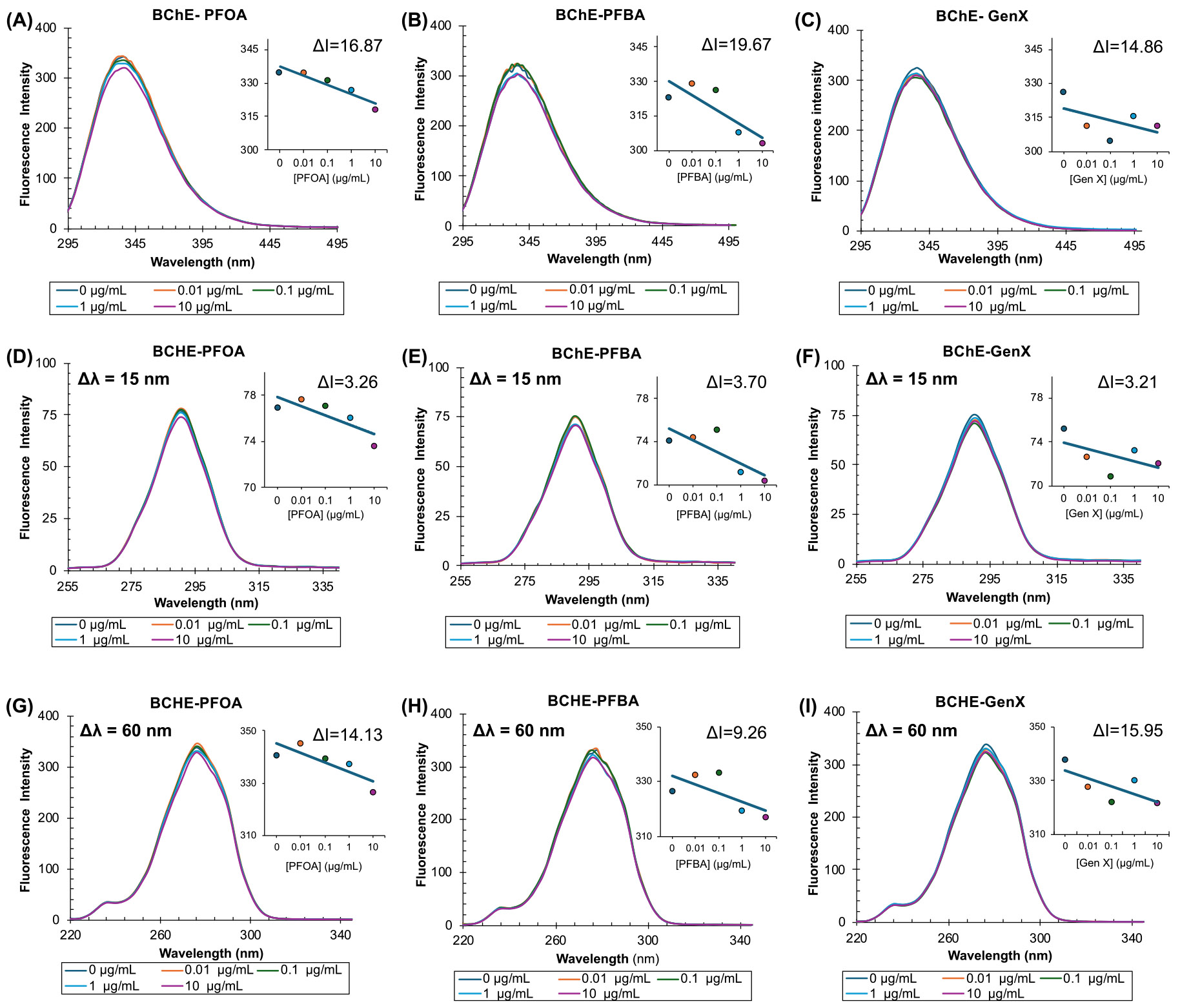
Fluorescence and synchronous fluorescence spectra of butyrylcholinesterase (BChE) in the absence and presence of PFAS. (A–C) Fluorescence emission spectra (*λ*_ex_ = 280 nm; *λ*_em_ = 336 nm) of BChE treated with (A) PFOA, (B) PFBA, and (C) GenX. (D–F) Synchronous fluorescence spectra (*λ*_ex_ = 250–345 nm (Δλ = 15 nm); *λ*_em_ = 292 nm) of BChE treated with (D) PFOA, (E) PFBA, and (F) GenX.(G–I) Synchronous fluorescence spectra (*λ*_ex_ = 220–345 nm (Δλ = 60 nm); *λ*_em_ = 275 nm) of BChE treated with (G) PFOA, (H) PFBA, and (I) GenX. Insets display scatter plots of fluorescence intensity as a function of PFAS concentration. Experiments were conducted at 25 °C and pH 8.0, with a BChE concentration of 0.2 mg/mL.

The intrinsic fluorescence and synchronous fluorescence spectra of AChE and BChE demonstrated differential quenching effects in response to treatment with PFOA, PFBA, and GenX (Figs. 5 and 6). Overall, a reduction in fluorescence intensity was observed across all treated samples compared to the untreated control. For comparative purposes, the extent of quenching was quantified as the difference in peak fluorescence intensity (ΔI) between the untreated sample and the sample treated with the highest test concentration (10 μg/mL PFAS).

In fluorescence spectra for AChE, PFOA caused the most significant reduction in fluorescence intensity (ΔI = 19.72), indicating the strongest quenching effect (Fig. 5). This was followed by GenX (ΔI = 19.50) and PFBA (ΔI = 10.61). Synchronous fluorescence spectra at Δλ of 15 nm, which primarily reflect changes in tyrosine residues, showed a distinct order of quenching. GenX induced the greatest quenching (ΔI = 4.25), followed by PFOA (ΔI = 3.12) and PFBA (ΔI = 1.13). At a Δλ of 60 nm, predominantly monitoring tryptophan residues, the order was reversed, with PFBA exhibiting the most potent quenching effect (ΔI = 23.19), followed by GenX (ΔI = 20.66) and PFOA (ΔI = 14.90).

For BChE, the conventional fluorescence measurements revealed that PFBA caused the most pronounced reduction in fluorescence intensity (ΔI = 19.67), followed by PFOA (ΔI = 16.87) and GenX (ΔI = 14.86) (Fig. 6). A similar trend was observed in synchronous fluorescence measurements at Δλ = 15 nm, with quenching intensities of 3.70, 3.26, and 3.21 for PFBA, PFOA, and GenX, respectively. However, at Δλ = 60 nm, a reversed pattern emerged: GenX exhibited the strongest quenching effect (ΔI = 15.95), followed by PFOA (ΔI = 14.13) and PFBA (ΔI = 9.26).

**Fig. 7.**
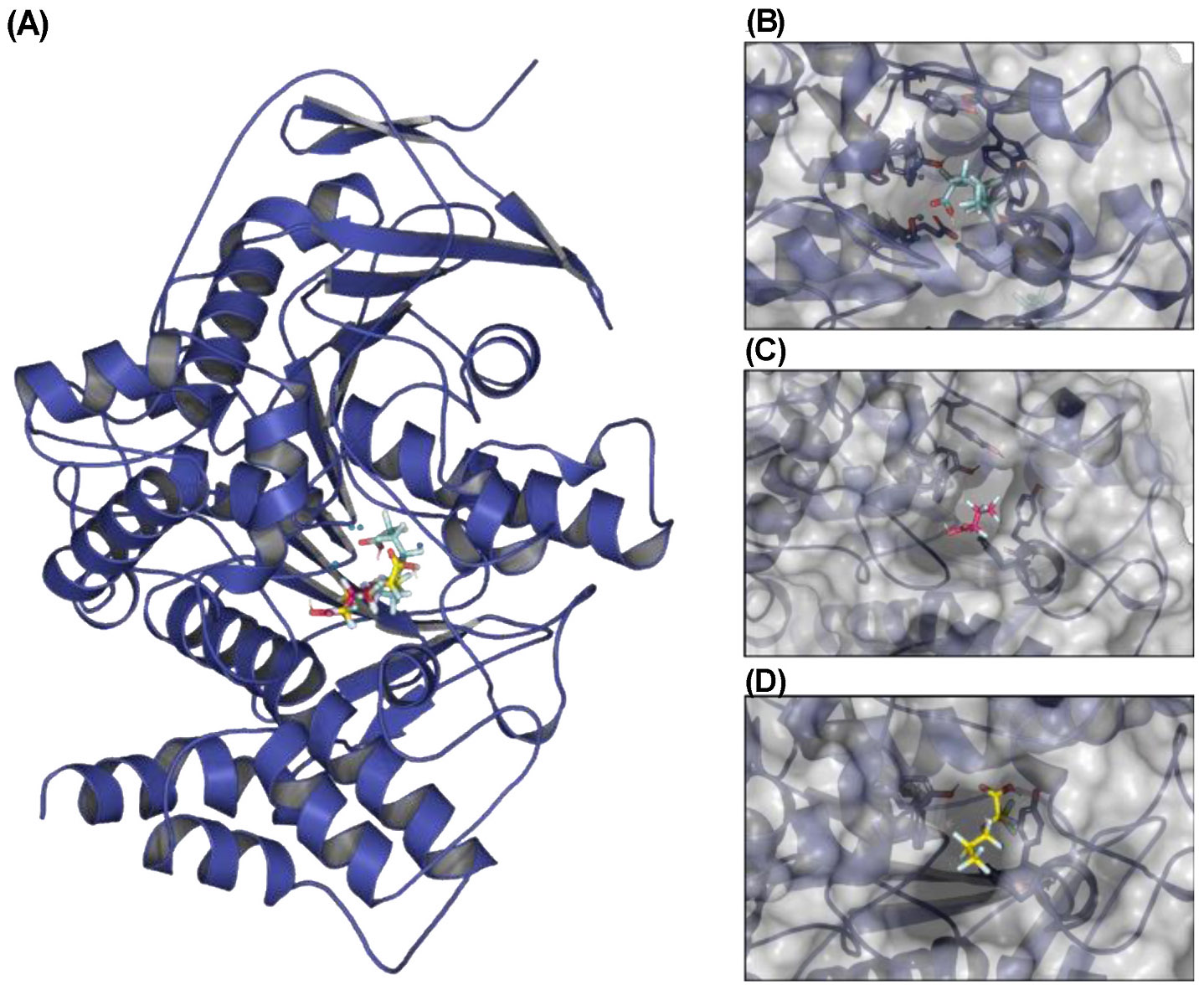
(A) The protein view of human acetylcholinesterase (PDB ID 4EY7) with the best binding poses of PFOA, PFBA, and GenX is depicted in cyan, pink, and yellow, respectively. (B–D) Close-up views of each ligand with the protein surface were modeled to visualize the binding pockets. The binding energies of PFOA, PFBA, and GenX were −9.806, −6.676, and −8.227 kcal/mol, respectively.

Four potential binding sites were produced by docking PFOA, PFBA, and GenX with AChE as the receptor (Fig. S9). However, not every molecule occupied each binding site. Binding for each molecule was strongest in the active site (Fig. 7), and binding energies for PFOA and GenX were within 1–2 kcal/mol of what has been previously reported for that of AChE and the inhibitor Donepezil (Table S2) (Hakeem, 2023). PFBA consistently showed weaker binding than PFOA and GenX for all sites. All possible binding sites identified through docking for AChE are found in Figures S9 through S12.

**Fig. 8.**
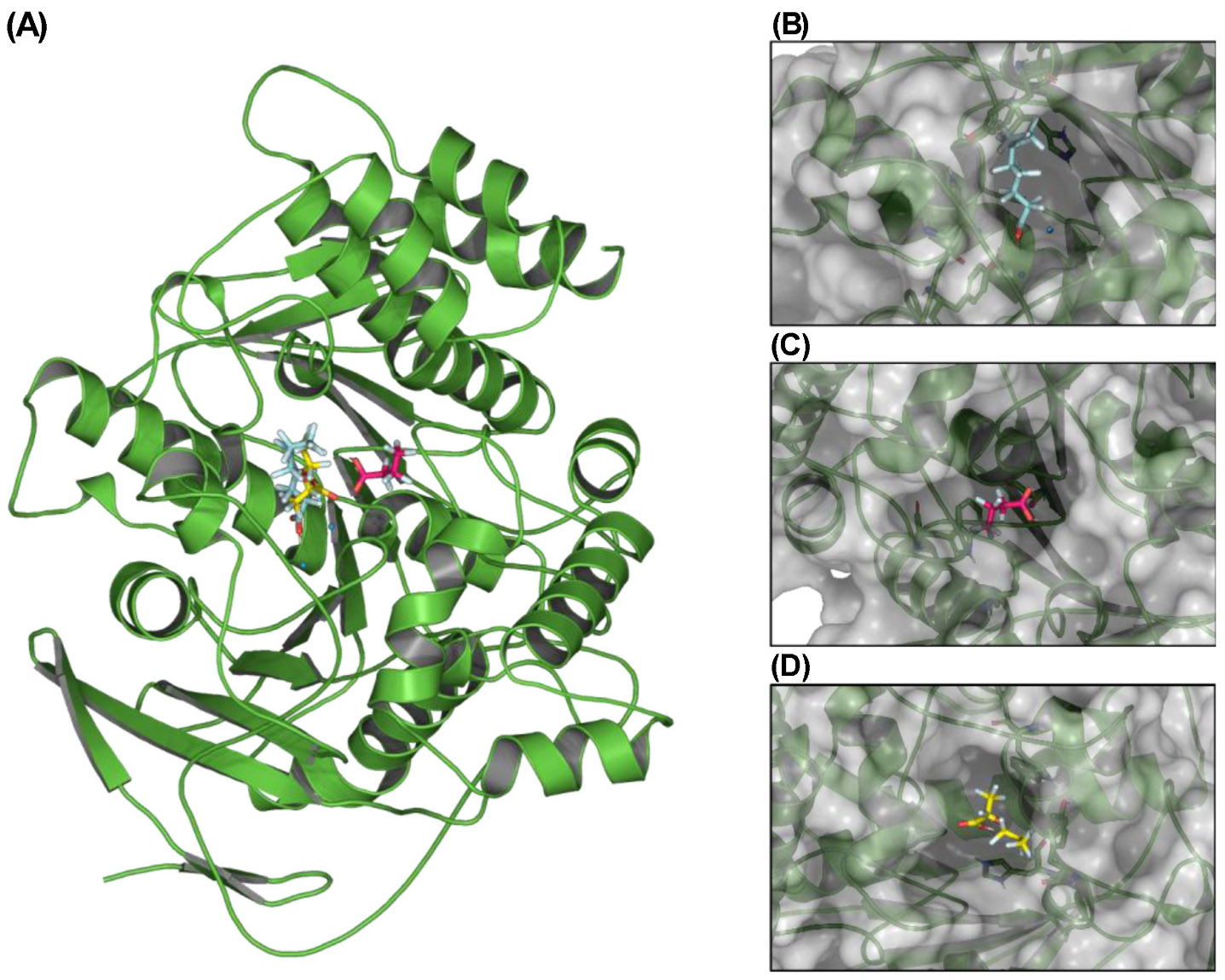
(A) The protein view of human butyrylcholinesterase (PDB ID 4TPK) with best binding poses of PFOA, PFBA, and GenX depicted as cyan, pink, and yellow, respectively. (B–D) Close-up views of each ligand with the protein surface were modeled to visualize the binding pockets. The binding energies of PFOA, PFBA, and GenX were −8.119, −6.442, and −7.077 kcal/mol, respectively.

PFOA, PFBA, and GenX docking with BChE identified seven potential binding sites. Docking for the strongest pose of each ligand is shown in Fig. 8. Again, not every ligand is bound to each site, and the active site exhibited the strongest binding for each ligand. Ligand binding energies for BChE were generally weaker overall than those in the inhibitor studies, except for PFOA (Table S3) (Williams et al., 2019). All possible binding sites identified through docking for BChE are included in Fig. S13-S14.

## 4. Discussion

This study employed a multifaceted approach to evaluate the impact of PFAS on neurotransmission. Our central hypothesis was that PFAS may disrupt key aspects of neurotransmission, including catecholamine release and cholinesterase activity. Studies have shown that the cholinergic and catecholaminergic systems are interconnected and impact each other. An imbalance between both is postulated to result in adverse consequences.

First, the effect of PFAS on neurotransmitter release was analyzed as a measure of cytotoxicity. Based on previous findings, the PFAS concentrations used in this study did not compromise cell viability (Obiako et al., 2024). Second, we assessed the impact of PFAS on the function of neurological enzymes in a human neuronal cell model (SH-SY5Y). SH-SY5Y cells were chosen as a suitable biological model for this work due to their extensive characterization and widespread use in studying neuronal function and the progression of neurodegenerative disorders. These cells exhibit neuronal-like morphology and express essential neurotransmitters, transporters, and neurological enzymes, making them suitable for investigating neurobiological processes (Feles et al., 2022). Third, biochemical analyses were conducted under cell-free conditions using spectroscopic techniques to investigate direct PFAS-cholinesterase interactions, aiming to elucidate potential mechanisms by which PFAS disrupts neurotransmission. Finally, molecular docking simulations were performed to complement experimental findings, providing insights into the structural and functional basis of PFAS-enzyme interactions.

Neurotransmission is crucial in assessing neurotoxicity, as neurotransmitters are vital for maintaining the functional integrity of both the central and peripheral nervous systems. These chemical messengers are essential for signal transmission between neurons and other cells, significantly influencing behavior, cognition, and overall neural homeostasis (Teleanu et al., 2022). Among neurotransmitters, catecholamines—a group of monoamines synthesized from tyrosine through hydroxylation and decarboxylation—hold particular importance due to their diverse physiological roles (Young, 2011; Rios et al., 1999). This group includes dopamine, norepinephrine (also known as noradrenaline), and epinephrine (also known as adrenaline), each serving distinct yet interconnected functions. Dopamine (4-(2-aminoethyl)-1,2-benzenediol) is integral to movement regulation, attention, cognition, motivation, and the sensation of pleasure; norepinephrine (noradrenaline), modulates emotional responses, sensory signal detection, focus, memory, learning, and sleep; and epinephrine (a. k.a. adrenaline) triggers the body’s fight-or-flight response (Wong et al., 2012; Tully et al., 2010; Ranjbar-Slamloo et al., 2019; O’Donnell et al., 2012). Given their pivotal roles, maintaining optimal levels of catecholamines is imperative for physiological and psychological well-being.

Our results align with prior studies on PFAS-induced neurotransmitter dysregulation, including catecholaminergic pathways, suggesting a possible link to neurodegenerative risks (Brown-Leung et al., 2022). While total catecholamine release was altered only in PFOA-treated cells, individual catecholamines exhibited differential responses. Dopamine release under depolarizing conditions was significantly increased across all PFAS-treated cells, highlighting a potential disruption of dopaminergic signaling pathways. Several experimental investigations using invertebrate and vertebrate animal models corroborate these findings, as PFAS exposure has been shown to adversely affect catecholaminergic systems. For instance, PFOS exposure in a nematode model increased levels of dopamine, serotonin, and γ-aminobutyric acid (GABA) (Yuan et al., 2018). Similarly, planarians exposed to PFOA showed increased dopamine concentrations after 4 days (Zhang et al., 2020). This pattern of neurotransmitter alterations extends to mammalian models, where PFOA-exposed mice exhibit heightened dopamine and serotonin levels alongside reduced norepinephrine concentrations (Yu et al., 2016).

Rodent studies further revealed that PFOS-induced region-specific dopamine increases, particularly in cognitive centers such as the prefrontal cortex and hippocampus (Salgado et al., 2016). In contrast, a study using an amphibian model demonstrated significant dopaminergic depletion following exposure to PFOA and PFOS (Foguth et al., 2019). Likewise, the concentrations of multiple neurotransmitters, including dopamine, noradrenaline, acetylcholine, glutamate, 5-hydroxytryptamine, and γ-aminobutyric acid, were significantly reduced in zebrafish larvae exposed to perfluorononanoic acid (PFNA) (Liu et al., 2023). Zhao et al. also found that exposure to PFHxA, PFOA, and PFOS at environmentally relevant concentrations resulted in decreased dopamine levels, with no significant changes in norepinephrine levels in fish brain tissues, which correlated with observed behavioral changes (Zhao et al., 2024). Moreover, PFOS exposure has been shown to induce dopaminergic neuron degeneration, highlighting the sensitivity of these neurons to PFAS toxicity (Sammi et al., 2019). Another new-generation PFAS, C_6_O_4_, which is commonly used alongside GenX in industrial applications, has also demonstrated neurotoxic potential. Di Nisio et al. investigated the effects of C_6_O_4_on human dopaminergic neurons derived from induced pluripotent stem cells. They reported minimal effects on cell membrane properties and cell molecular phenotype after a 24 h exposure to 10 ng/mL C_6_O_4_ (Di Nisio et al., 2023). These findings, together with ours, highlight the need for further comparative studies to understand better structure-neurotoxicity relationships within this broad chemical class, particularly among new-generation PFAS.

While a moderate increase in dopamine levels enhances functions like motivation and cognition, its dysregulation has been implicated in a spectrum of psychiatric and neurological conditions like schizophrenia and Alzheimer’s, Parkinson’s, and Huntington’s diseases (Teleanu et al., 2022; Speranza et al., 2021). Dopamine exhibits an inverted-U relationship with cognitive performance, where both insufficient and excessive dopamine can impair cognitive function (Weber et al., 2022; Cools et al., 2011). Elevated dopamine levels, in particular, have been associated with increased oxidative stress due to the production of reactive oxygen species (ROS) and dopamine quinones, which are hypothesized to be the underlying mechanisms of dopamine-mediated neurotoxicity (Kita et al., 2009). Additionally, excessive dopamine in animal brains has been shown to cause cognitive impairment, potentially due to overstimulation of dopamine receptors (Murphy et al., 1996). This overstimulation can disrupt neural signaling and impair synaptic plasticity, critical learning, and memory processes (Scheler, 2004; Klein et al., 2019; Goto, 2022).

Significant alterations in norepinephrine release were observed only in PFOA-treated cells. As a derivative of dopamine, norepinephrine plays a crucial role in enhancing cognitive function, vigilance, focus, and motor performance. However, excessive norepinephrine levels can have toxicological implications, including cardiovascular dysregulation (*e.g.*, hypertension, tachycardia), renal impairment, sleep disturbances, anxiety, and cognitive deficits (Jordan et al., 2018; Holland et al., 2021). Compared to controls, the release of adrenaline was significantly reduced in cells treated with PFOA, PFBA, and GenX. Adrenaline, synthesized from noradrenaline via phenylethanolamine N-methyltransferase (PNMT), is crucial for mediating stress responses (Wong et al., 2012; Drinkwater et al., 2009). Reduced adrenaline levels have been linked to lower blood pressure and deficits in memory and learning (Wong et al., 2012; Pravosudov, 2019).

The maintenance of healthy synaptic communication critically depends on precise neurotransmitter clearance mechanisms, including enzymatic degradation by cholinesterases (Perdan et al., 2009; Hyman, 2005). Acetylcholinesterase (AChE) and butyrylcholinesterase (BChE) terminate nerve impulse transmission by catalyzing the hydrolysis of choline-based neurotransmitters, such as acetylcholine (Hyman, 2005; Masson et al., 2010). Notably, cholinesterase inhibition has been implicated in the neurotoxicity associated with organophosphates, a class of compounds commonly found in pesticides and chemical warfare agents (Masson et al., 2010; Nichols et al., 2021).

Our investigation revealed differential effects of PFAS compounds on cholinesterase activities across cellular and cell-free experiments. When examining the overall enzyme activity values of PFAS-treated samples relative to the untreated control group, AChE exhibited a general trend of decreased activity in both cellular and cell-free conditions. Interestingly, BChE showed elevated activity relative to the untreated control group, with effects most pronounced and statistically significant in cell-free assays. This opposing effect on the two cholinesterases is particularly striking and warrants further investigation to clarify the underlying mechanisms. The observed AChE inhibition has profound physiological implications, given the enzyme’s critical role in cholinergic neurotransmission. Inhibition of AChE activity can lead to the accumulation of acetylcholine in the brain and neuromuscular junctions, potentially inducing a cholinergic crisis. This crisis manifests through muscarinic effects, such as miosis, increased secretions (salivation, lacrimation), diarrhea, urination, and nicotinic effects, including muscle fasciculations and neuromuscular blockade (Colovic et al., 2013). In severe cases, these symptoms can escalate to respiratory failure and death (Colovic et al., 2013; Nervo et al., 2019).

The detected increase in BChE activity is concerning, given its association with heightened inflammatory responses and an increased risk of cognitive disorders (Hughes et al., 2022). BChE has been shown to act as a compensatory enzyme for AChE, particularly due to stress or neurotoxicity (Li et al., 2022; Khan et al., 2023). BChE is also increasingly recognized as a biomarker in neurodegenerative diseases such as Alzheimer’s disease (Macdonald et al., 2017). Limited studies have associated PFAS exposure to changes in cholinesterase activity, particularly AChE. The observed inhibition of AChE activity following PFAS exposure is consistent with results from prior investigations. Studies have reported a significant decline in AChE activity in planarians exposed to PFAS (Yuan et al., 2018; Zhang et al., 2020). Similarly, Zhao et al. noted that exposure to environmentally relevant concentrations of PFOA and PFOS led to reduced AChE activity in the brain tissues of fish, which was suspected to cause behavioral changes (Zhao et al., 2024). Liu et al. observed that AChE and cholinesterase (ChE) activities were significantly reduced in zebrafish larvae exposed to perfluorononanoic acid (PFNA) (Liu et al., 2023). Alterations in the transcription of AChE in PFOS-treated mice and fish have also been reported, suggesting that the effects on AChE may be beyond the activity level (Hallgren et al., 2015; Khazaee et al., 2020).

The occurrence of schizophrenia and Parkinsonism has been linked to disturbances in the balance between the cholinergic and dopaminergic systems, and neuropharmacological strategies aimed at managing these conditions often target restoring this neurochemical balance (Acharya et al., 2021; Amalric et al., 2021; Vaiman et al., 2022; Scarr et al., 2013). The cholinergic system, primarily mediated by acetylcholine, has been shown to modulate dopaminergic activity. Recent studies have reported that acetylcholine triggers dopamine release, particularly in the striatum (Liu et al., 2022; Chantranupong et al., 2023). This study observed AChE inhibition, which is directly associated with increased acetylcholine levels (Colovic et al., 2013). Elevated acetylcholine can enhance its modulatory effects on dopamine signaling, potentially leading to disruptions in neurotransmitter dynamics. These findings suggest that PFAS exposure may perturb cholinergic-catecholaminergic interactions, contributing to neurotoxic outcomes. Understanding such interactions is crucial for elucidating the neurotoxicity associated with PFAS.

^19^F NMR spectroscopy is a powerful and selective analytical tool extensively used to investigate binding interactions between PFAS and proteins (Fedorenko et al., 2021; D’Eon et al., 2010; Rand et al., 2012; MacManus-Spencer et al., 2010; Liu et al., 2019). This technique offers distinct advantages due to the characteristic chemical shifts from the perfluorocarbon moiety and the absence of interfering background signals from the protein matrix (Camdzic et al., 2021). The binding events between PFAS and the enzymes were effectively monitored through observable spectral perturbations, specifically manifested as chemical shift variations and peak broadening of the fluorine resonance signals.

The ^19^F NMR spectral analysis revealed that PFOA exhibited the strongest binding interaction with AChE and BChE, as evidenced by substantial chemical shift changes and peak broadening of the α and ω fluorine groups. These effects were most evident at the PFAS:enzyme ratio of 2:1, indicating the formation of PFOA-cholinesterase complexes. The observed spectral changes suggest that the chemical environment of PFOA changed significantly upon interaction with the enzymes, possibly due to its incorporation into their binding sites. In contrast, PFBA and GenX exhibited minimal or negligible spectral changes under identical conditions, indicating weaker or insignificant binding interactions with the enzymes. This differential binding behavior may be attributed to PFOA’s longer carbon chain and larger surface area, which likely promotes stronger hydrophobic interactions with proteins (Bangma et al., 2022). In contrast, PFBA’s shorter chain and GenX’s branching structure may limit their interaction possibilities.

Enzyme function is closely tied to structural and conformational integrity, so alterations in these properties can lead to physiological dysfunction (Lavery et al., 2007). Fluorescence spectroscopy is a valuable tool for examining the structure and conformation of proteins and elucidating the interactions between proteins and small molecules. It provides insights into molecular binding parameters such as mechanisms, modes, sites, constants, and intermolecular spatial relationships (Xiang et al., 2009). Proteins inherently exhibit strong fluorescence emission due to the presence of aromatic amino acid residues, primarily tryptophan (Trp), tyrosine (Tyr), and phenylalanine (Phe) (Xu et al., 2018). A decline in fluorescence intensity, referred to as fluorescence quenching, is often observed during such interactions and can arise from various molecular processes, including energy transfer, molecular rearrangements, excited-state reactions, ground-state complex formation, and collisional quenching (Xiang et al., 2009).

The fluorescence and synchronous fluorescence spectra analyses reveal unique interactions between PFAS compounds (PFOA, PFBA, and GenX) and the cholinesterase enzymes (AChE and BChE), indicating compound and enzyme-specific interactions. The consistent quenching of fluorescence intensity across all treated samples relative to untreated controls highlights the ability of the tested PFAS compounds to alter the microenvironment of cholinesterase enzymes and specific amino acid residues within these enzymes, potentially through direct binding or conformational changes. The differential quenching effects observed between AChE and BChE, as well as the variations observed in the synchronous fluorescence spectra, indicate that PFOA, PFBA, and GenX may have different binding mechanisms and preferential interaction sites within the cholinesterase enzymes, reflecting varying modes of interaction.

However, circular dichroism (CD) data (Fig. S8) indicate no significant alterations in the secondary structures (α-helical and β-sheet content) of both AChE and BChE upon treatment with the tested PFAS. This implies that the observed fluorescence quenching is likely a result of changes in the enzymes’ local environment caused by interactions or binding between PFAS and fluorophores rather than any alterations in the enzymes’ secondary structure or global conformation.

Molecular docking simulations provide valuable insights that complement the findings from ^19^F NMR spectroscopy and fluorescence spectroscopy, further elucidating the interactions between PFAS and cholinesterase enzymes. Simply put, the NMR data indicate that it binds, and the fluorescence data and the docking provide insights into where the binding occurs. The simulations revealed that PFOA preferentially binds to the deep binding pockets of AChE, achieving lower binding energies than its shorter-chain counterparts. This deep-pocket interaction of PFOA correlates with the significant chemical shift changes and peak broadening observed in NMR spectra, validating the formation of enzyme-PFAS complexes. In contrast, the shorter-chain PFAS compounds were bound to shallow binding pockets with notably higher binding energies, consistent with their minimal NMR spectral perturbations. The lower signal in higher concentrations of PFAS is likely due to PFAS excess, where more unbound PFAS are present in solution as the binding sites of the cholinesterase enzymes are saturated. This trend was also observed in BChE, where PFOA demonstrated the lowest binding energy among the tested PFAS compounds. These findings suggest that PFAS chain length and structural properties govern their binding affinity and localization within protein binding sites (Bangma et al., 2022).

From the molecular docking results of AChE, Trp 36, Tyr 337, and Tyr 341 were identified as fluorescently active residues close enough to the docked ligands in the active site to experience an altered electronic environment upon ligand binding. Likewise, for BChE, Trp 430, Tyr 332, Trp 82, Trp 231 were identified as residues whose fluorescence may be altered upon ligand binding. This alteration of fluorescence is consistent with the results observed in the synchronous fluorescence section. It suggests effects on conformation upon binding of these ligands to their respective enzyme in the identified sites. Together with the binding energies observed in this study for AChE and BChE, which are comparable to those in previous inhibitor studies with the same enzymes (Williams et al., 2019), these docking results support the notion that PFOA, PFBA, and GenX interact with AChE and BChE in potentially function-altering effects.

## 5. Conclusion

This study proves that PFAS, specifically PFOA, PFBA, and GenX, can dysregulate neurotransmission pathways through multiple mechanisms. Our findings reveal altered catecholamine neurotransmitter release in PFAS-exposed neuronal cells, further corroborated by modulations in cholinesterase (AChE and BChE) function. Additionally, evidence shows that the examined PFAS interact uniquely with cholinesterases, which may explain the observed change in cholinesterase activity. These observed disruptions have the potential to cascade into synaptic miscommunication and subsequent neuronal impairment. These findings contribute to a growing body of evidence on the neurotoxic potential of PFAS and underscore the need for further investigations to elucidate the molecular mechanisms underlying PFAS-induced neurotoxicity, particularly given the widespread presence and persistence of these chemicals.

## Supplementary Material

1

## Figures and Tables

**Table 1:** The concentrations of PFAS, PFBA, and GenX used in this study.

PFAS	Mass per volume	Molarity
***PFOA***	0.01–1 μg/mL	0.0242–2.42 μM
***PFBA***	0.01–1 μg/mL	0.0467–4.67 μM
***GenX***	0.01–1 μg/mL	0.0303–3.03 μM

## Data Availability

Data will be made available on request.

## HIGHLIGHTS

- PFOA, PFBA, and GenX can disrupt neurotransmission pathways by various mechanisms.
- Changes in cholinesterase function and catecholamine release occurred after PFAS exposure.
- Each PFAS uniquely interact with cholinesterases, which explains the change in activity.
- PFOA, PFBA, and GenX are potentially neurotoxic.

## Appendix A. Supplementary data

Supplementary data to this article can be found online at https://doi.org/10.1016/j.chemosphere.2025.144678.
