# Supplementary material for: Perfluorooctanoic acid, perfluorobutanoic acid, and undecafluoro-2-methyl-3-oxahexanoic acid disrupt neurotransmitter release and cholinesterase activity
Source: Chemosphere. Author manuscript; Available in PMC 2026 Jun 23. (PMC13287238; doi:10.1016/j.chemosphere.2025.144678)

*Supporting Information of*

**TITLE**

Perfluoroalkyl substances alter neurotransmitter release and enzymatic dynamics

**AUTHORS**

Precious C. Obiako^1^, Bryan Taylor^2^, Jonathan Clinger^2^, Christie M. Sayes^1*^

**AFFILIATIONS**

^1^Department of Environmental Science, Baylor University, Waco, TX 76798, United States of America

^2^Department of Chemistry, Baylor University, Waco, TX 76798, United States of America

**CORRESPONDING AUTHOR**

Christie Sayes; Department of Environmental Science, Baylor University, One Bear Place #97266, Waco, TX 76798-7266, USA. Email: [Christie_sayes@baylor.edu](mailto:Christie_sayes@baylor.edu)

This supplementary material contains 3 tables and 14 figures.

**Table S1.** Physicochemical characteristics of the three perfluoroalkyl substances used in this study.


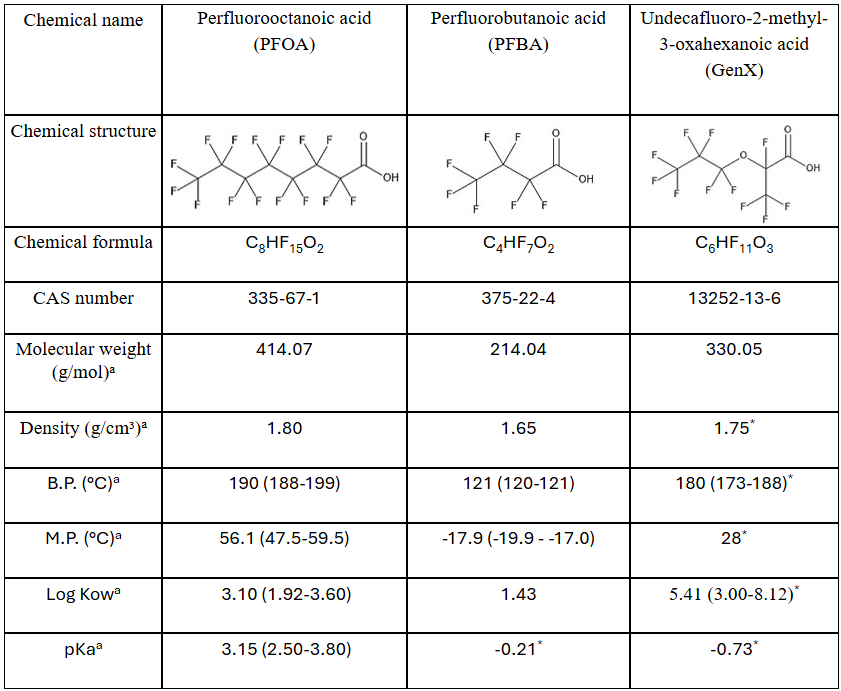
^a^The information was compiled from U.S. EPA CompTox Chemicals Dashboard

^*^ Predicted value

***Figure S1.*** *Absorbance graphs depicting acetylcholinesterase (AChE) activity in SH-SY5Y cells upon exposure to (A) PFOA, (B) PFBA, and (C) GenX. Absorbance was measured at 412 nm over time, and the calculated slopes were utilized to determine relative AChE activity.*

***Figure S2.*** *Absorbance graphs depicting butyrylcholinesterase (BChE) activity in SH-SY5Y cells upon exposure to (A) PFOA, (B) PFBA, and (C) GenX. Absorbance was measured at 412 nm over time, and the calculated slopes were utilized to determine relative BChE activity.*

******

***Figure S3.*** *Absorbance graphs depicting acetylcholinesterase (AChE) activity in cell-free conditions following treatment with (A) PFOA, (B) PFBA, and (C) GenX. Absorbance was measured at 412 nm over time, and the calculated slopes were utilized to determine relative AChE activity.*

***Figure S4.*** *Absorbance graphs depicting butyrylcholinesterase (BChE) activity in cell-free conditions following treatment with (A) PFOA, (B) PFBA, and (C) GenX. Absorbance was measured at 412 nm over time, and the calculated slopes were utilized to determine relative BChE activity.*

**

***Figure S5.*** *^19^F-NMR spectra of (A) PFOA, (B) PFBA, and (C) GenX, with assigned peaks labeled.*

***Figure S6.*** *UV–vis absorption spectra of acetylcholinesterase (AChE) in the absence and presence of (A) PFOA, (B) PFBA, and (C) GenX.*

***Figure S7.*** *UV–vis absorption spectra of butyrylcholinesterase (BChE) in the absence and presence of (A) PFOA, (B) PFBA, and (C) GenX.*

***Figure S8.*** *Circular dichroism (CD) spectra of cholinesterases upon treatment with PFAS. (A) CD spectra of acetylcholinesterase (AChE) and (B) CD spectra of butyrylcholinesterase (BChE) in the absence (control) and presence of 10 µg/mL PFOA, PFBA, and GenX. Data was normalized to molar ellipticity. Experiments were conducted at 25°C and pH 8.0, with cholinesterase concentrations maintained at 0.2 mg/mL.*

***Table S2.*** *AChE Ligand Binding Energies for Each Binding Site Determined via Docking*

|  | Ligand  (kcal/mol) | | |
| --- | --- | --- | --- |
| Site | PFOA | PFBA | GenX |
| 1 (deep) | -9.806 | -6.51 | N/A |
| 1 (shallow) | N/A | -6.676 | -8.227 |
| 2 | -7.641 | -5.478 | -7.218 |
| 3 | -6.53 | N/A | N/A |
| 4 | N/A | -6.383 | -7.503 |

***Table S3.*** *BChE Ligand Binding Energies for Each Binding Site Determined via Docking*

|  | Ligand  (kcal/mol) | | |
| --- | --- | --- | --- |
| Site | PFOA | PFBA | GenX |
| 1 | -7.629 | -6.442 | -6.823 |
| 2 | -8.119 | -5.898 | -7.077 |
| 3 | N/A | -6.052 | -6.721 |
| 3.2 | -7.654 | N/A | N/A |
| 4 | N/A | -6.365 | -6.328 |
| 5 | -6.669 | -5.935 | -6.869 |
| 6 | N/A | N/A | -6.72 |

***Figure S9.*** *(A) Whole protein view of human acetylcholinesterase (PDB ID 4EY7) with arrows pointing to ligand binding sites identified via docking. Cyan, pink, and yellow colors correspond to ligands PFOA, PFBA, and GenX. (B – C) Close-up views of PFOA binding in sites 1, 2, and 3, respectively.*


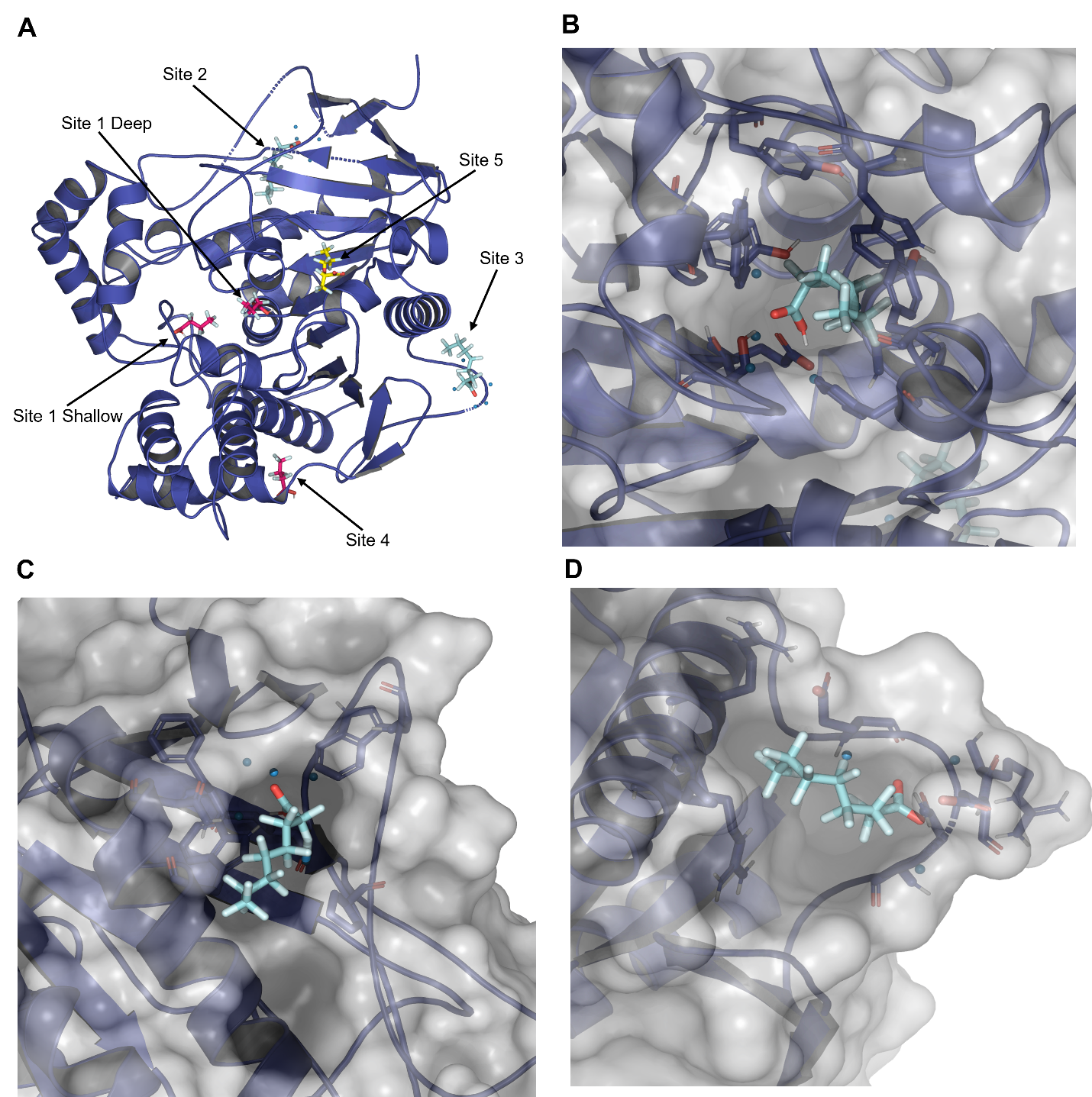


***Figure S10.*** *(A – E) Close-up views of PFBA binding sites identified by docking, corresponding to sites 1, 2, 3, 4, and 5, respectively.*


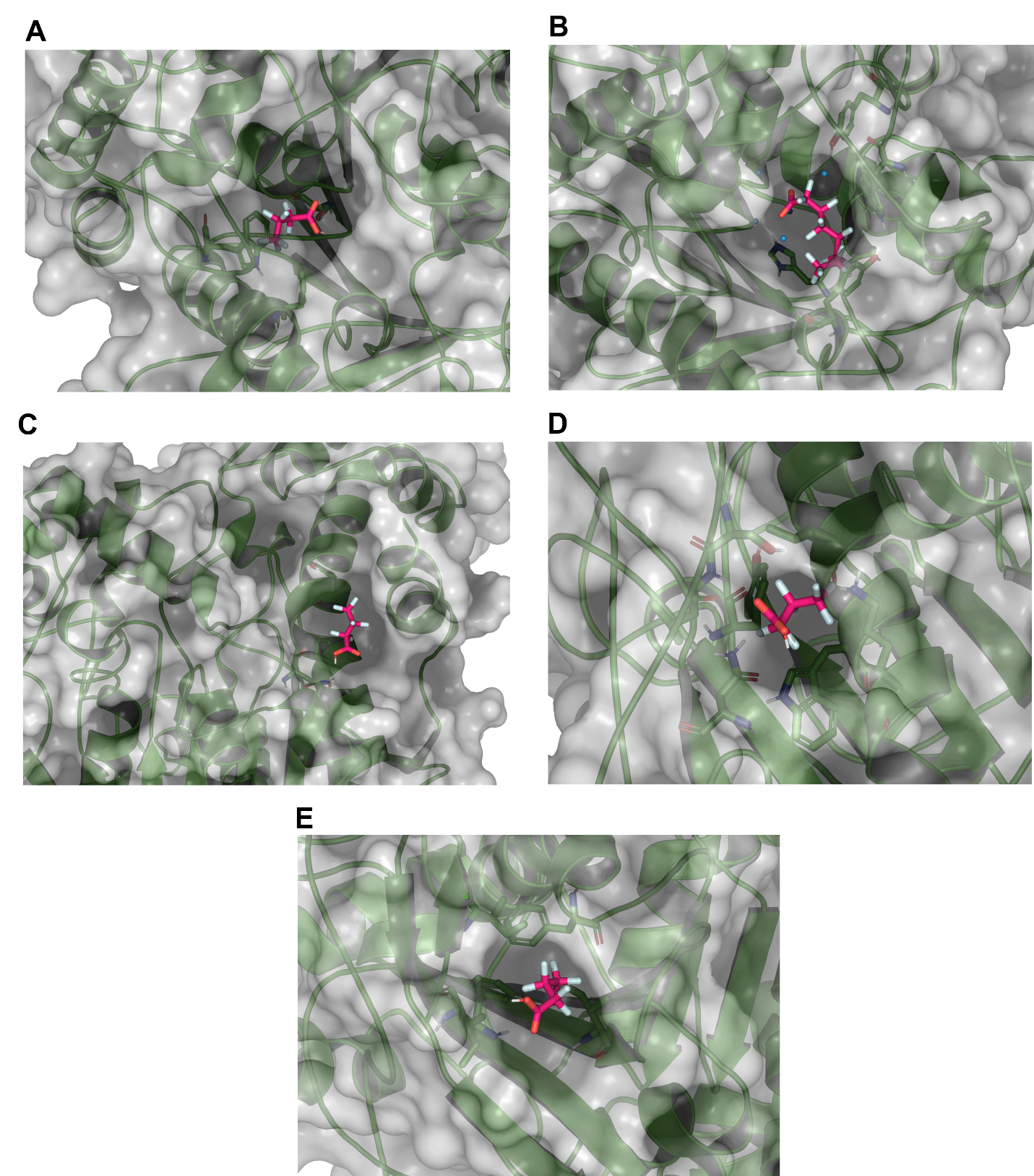


***Figure S11.*** *(A – D) Close-up views of PFBA binding sites identified by docking, corresponding to sites 1 Shallow, 1 Deep, 2, and 4, respectively.*


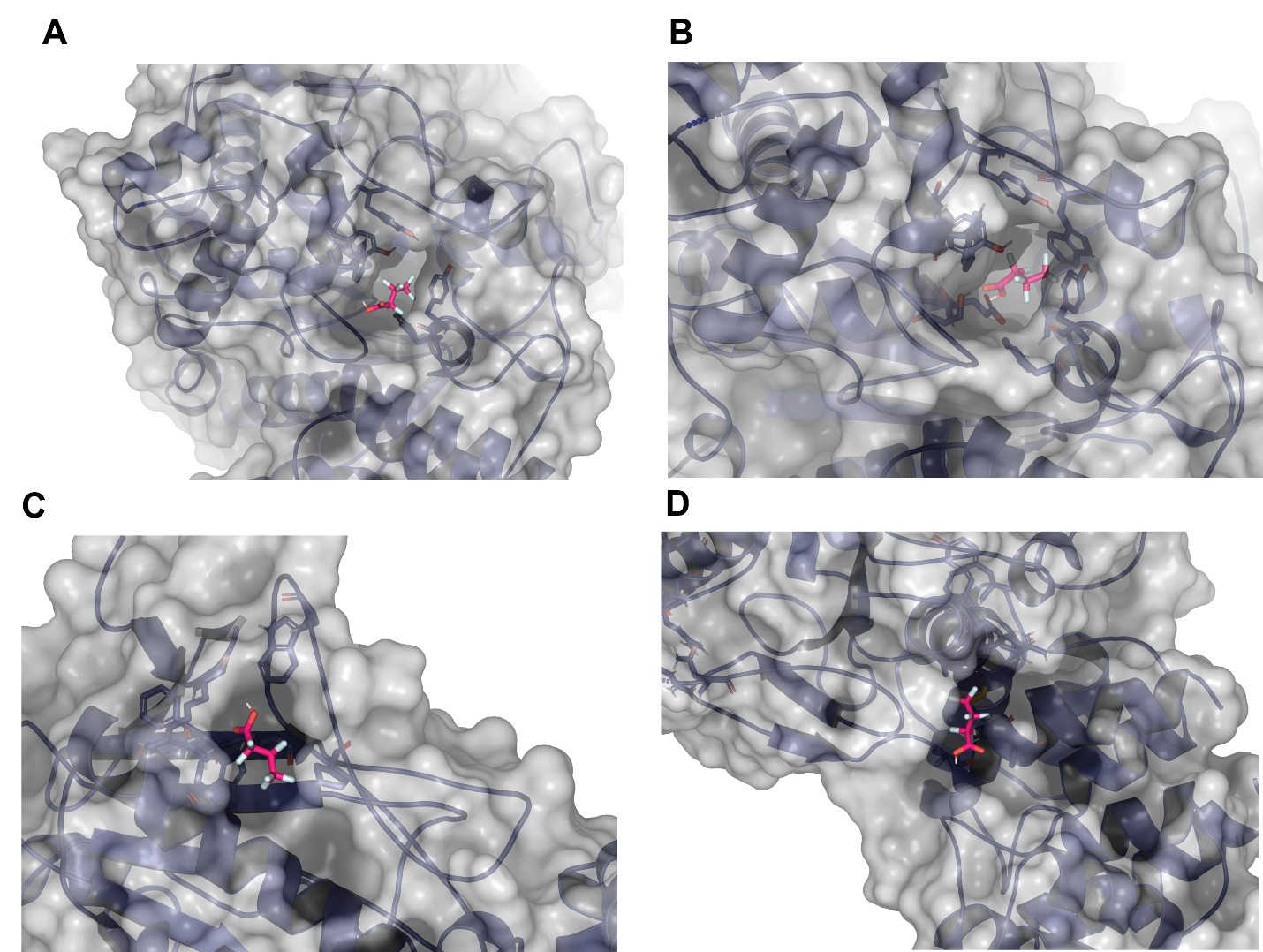


***Figure S12.*** *(A – C) Close-up views of GenX binding sites identified by docking, corresponding to sites 1, 2, and 4, respectively.*


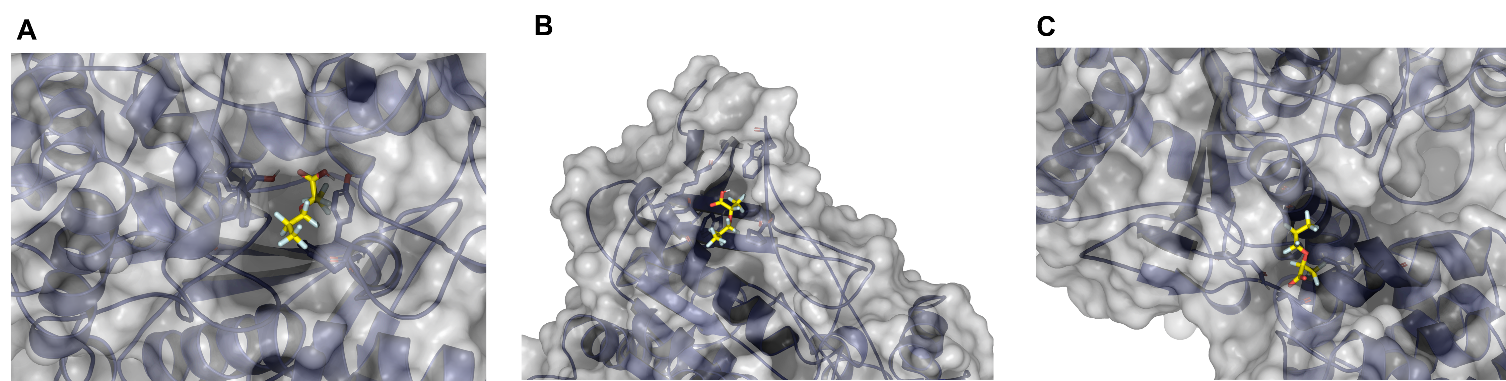


***Figure S13.*** *(A) Whole protein view of human butyrylcholinesterase* *(PDB ID 4TPK) with arrows pointing to ligand binding sites identified via docking. Cyan, pink, and yellow colors correspond to ligands PFOA, PFBA, and GenX. (B – E) Close-up views of PFOA binding in sites 1, 2, 3.2, and 5, respectively.*


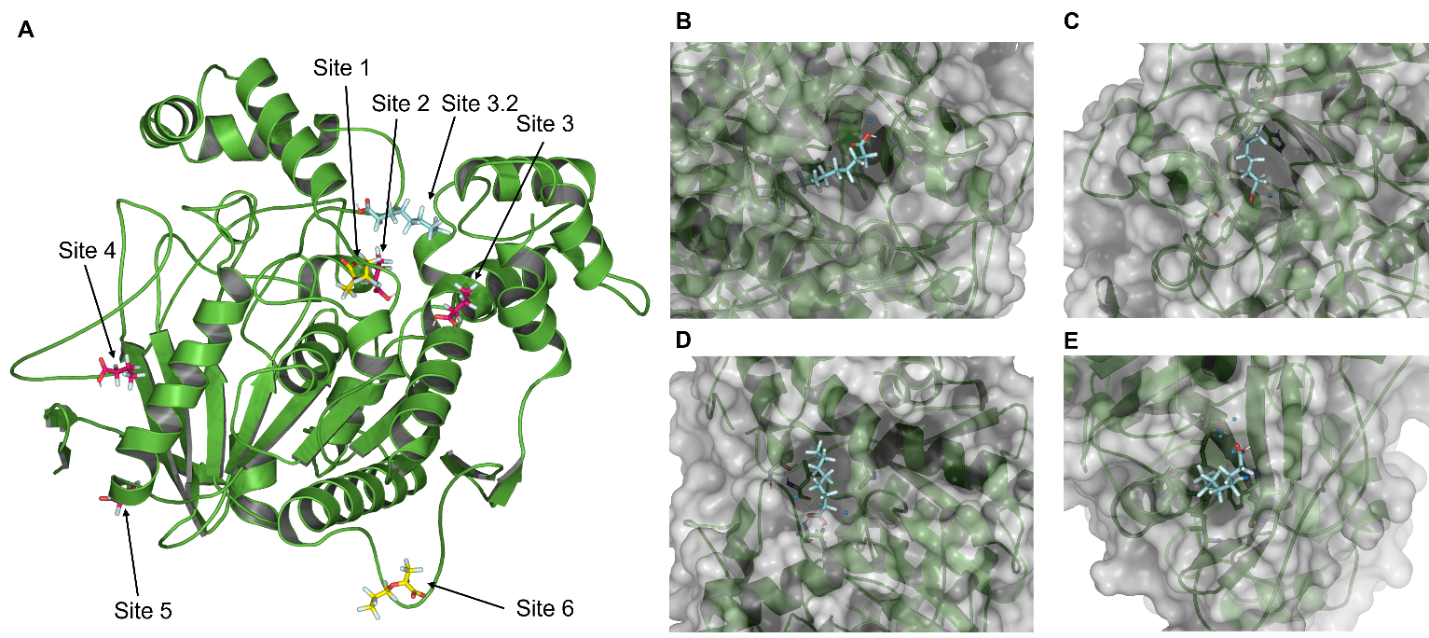


***Figure S14.*** *(A – F) Close-up views of GenX binding sites identified by docking, corresponding to 1, 2, 3, 4, 5, and 6, respectively.*


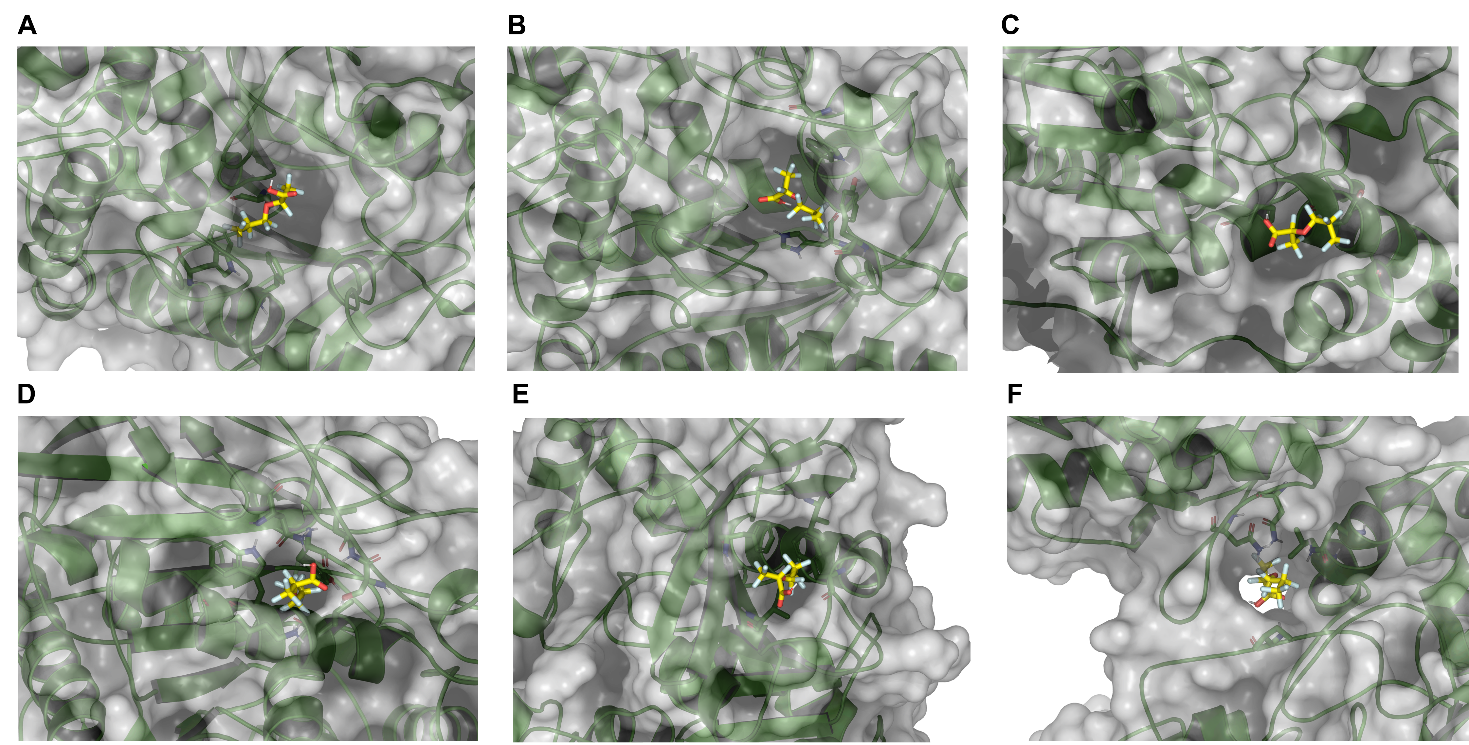

Supplement: 1 [file NIHMS2182394-supplement-1.docx]
